# Institutional Notes and News

**Published:** 1910-12-17

**Authors:** 


### INSTITUTIONAL NOTES AND NEWS.

**"Bethlehem" at Hampstead.**

Connoisseurs of fine art have a rare chance at Hampstead next week. On Tuesday and Wednesday, December 20 and 21, a Nativity play is to be given in Rosslyn Hill Chapel Room, Willoughby Road, Hampstead, at 5 o'clock, by the Hampstead Hospital Fairies. The proceeds will go to the Fairies' fund for the Hampstead Hospital. Tickets must be obtained *beforehand* from Mrs. Gow, 3 Keats' Grove, Hampstead. Anyone who had the joy of attending the two performances of the same play last year will remember that this entertainment differs from most in two respects—first, that the play is a genuine work of art; and, secondly, that the child actors do perfectly what our professional members have never learnt to do at all—that is, to speak English verse as verse, and not as prose made bad with blank lines at frequent intervals. The play is an adaptation of Laurence Housman's famous "Bethlehem," with Joseph Moorat's music, and is full of exquisite songs, of which the first, "The World is old to-night," is one of the most romantic and beautiful. All intelligent enough to take this opportunity of listening to "Bethlehem" not only will help the hospitals, but will see and hear acting and poetry to be seen and heard of equal quality nowhere else in London just now. The play will be preceded by Old English carols sung in costume. The seats are 2s. 6d. and 1s. 6d. respectively.

**Medical Women at Manchester.**

At a meeting of the board of the Manchester Royal Infirmary, held on December 6, the following scheme for the post-graduate education of medical women prepared by a special sub-committee was approved. The committee was appointed on March 1 last, and reported that it would be possible to create, for qualified medical women, one or more non-resident posts to be called "qualified clinical assistantships," which would provide valuable experience for the officer elected. The chief particulars are as follows: (1) Qualified medical women, who have been students at the Manchester Royal Infirmary throughout their course, shall be eligible for election to clinical assistantships, tenable for six months on the recommendation of the Medical Board. (2) They shall be appointed for merit only, and shall have no prescriptive right. (4) Their work shall be in the women's wards on the medical and surgical sides for two months each, and in the wards of the special departments for two months. (5) They shall work with, and under the immediate direction of, the resident medical or resident surgical officers. In the medical and surgical units their official duties shall be confined to the female wards. (6) During the two months they work in the special departments they shall assist, if required, in the out-patient department and at operations. (8) They shall live as near as practicable to the Infirmary, and provide their own board and lodging. (9) They shall receive a honorarium of £35 for the six months. (10) When in the Infirmary, but not on duty, they shall occupy the women students' room. The appointments of anæsthetist and assistant director of the clinical laboratory are reported as well open to qualified medical women.

**Cumberland Infirmary.**

On September 17 last, page 749, The Hospital gave a summary of the report which the Augmentation of Income Committee had presented at the quarterly meeting of the Cumberland Infirmary. Since then another quarter has passed, and we wish to call attention to the efforts that are being made to increase the revenue of an institution which accepts patients from both sides of the Tweed. Dr. Barnes in a capital speech summarised these efforts: Over 15,000 appeals have been sent out, 5,000 by the King Edward Memorial Committee, and 10,000 by the working-men of Carlisle. The latter are taking a keen interest in the Infirmary, and groups of them have been conducted over it during the past few months. This brings home to the imaginations of people who are well the constant work which goes on from day to day, and, we are sure, will be an immense encouragement to them and their fellows. The appeals have produced so far £1,850, which shows every sign of increasing rapidly in the next few weeks. We feel sure that this policy, so happily started, will bring home to those classes from which most of the Infirmary's patients come a sense of civic responsibility towards the hospital. At the end of the next quarter we hope, and expect, to hear of the continued and increased success of this appeal.

**The Birmingham Appeal.**

We are glad that, in spite of the distraction of an election, Birmingham has not forgotten the claims of its General Hospital, which we summarised on page 329 of The Hospital of last week. It is only fair that the public should be reminded that, apart from its concrete needs, the Birmingham General Hospital has another claim on their support. The appeal has been twice postponed, for its proposed issue early in the year was, of course, prevented by the death of the late King. When action again was contemplated the appeal of another hospital occupied the public mind; and good-fellowship and institutional solidarity alike once more had to acquiesce in a postponement. Now at last that the appeal is launched the General Election sweeps every other interest off the field. These arguments may well supplement the more concrete ones that we have published before. They have all the force of stern reality, but the public, nine times out of ten, knows nothing of such intangible difficulties. We hope that Birmingham will thank us for this peep behind the scenes by redoubling its efforts on behalf of the hospital, so that Christmas may not pass without it being known that the General Hospital Appeal has suffered nothing from difficulties which are, like the weather and the election, impossible to foresee and to avoid.

